# Development of a Medical Student Cardiopulmonary Resuscitation Elective to Promote Education and Community Outreach

**DOI:** 10.7759/cureus.4507

**Published:** 2019-04-20

**Authors:** Ashish Panchal, Samuel Keim, Gordon Ewy, Karl Kern, Kate E Hughes, Daniel Beskind

**Affiliations:** 1 Emergency Medicine, Ohio State University, Columbus, USA; 2 Emergency Medicine, Banner University Medical Center, Tucson, USA; 3 Cardiology, Banner University Medical Center, Tucson, USA; 4 Emergency Medicine, University of Arizona, Tucson, USA

**Keywords:** medical education, resuscitation research, cardiopulmonary resuscitation (cpr)

## Abstract

Introduction: One of the barriers to improving cardiac arrest survival is the low rate of cardiopulmonary resuscitation (CPR) provision. Identifying this as a public health issue, many medical students often assist in training the community in CPR. However, these experiences are often short and are not associated with structured resuscitation education, limiting the student’s and the community's learning. In this assessment, we identified a need and developed a curriculum, including defined goals and objectives, for an undergraduate medical education (UME) elective in CPR.

Methods: At an academic university environment with a strong UME program, we developed a longitudinal UME elective in CPR. The curriculum is a four-year longitudinal experience, which satisfies two weeks of their fourth year of medical school. The curriculum includes structured training over the four-year period in the fundamentals of resuscitation science (through didactics, journal club, and hands-on skills training), in addition to structured community CPR teaching. The elective concludes with a final hands-on summative appraisal. Data concerning medical student program enrollment, CPR training events conducted, venues of events, and the number of individuals trained were collected over a five-year period.

Results: The CPR elective was developed with clear goals and objectives based on identified needs. Over the five-year period, 186 medical students completed the CPR longitudinal elective, accounting for 8.4% of the total medical student population. Students completed curriculum requirements and satisfied both didactic and hands-on training with all students passing the final summative appraisal. Over the five-year period, students trained 8,694 people in bystander CPR. The summative evaluation had a 100% pass rate.

Conclusion: Implementation of a longitudinal CPR elective improved resuscitation science education for medical students and fostered increased community CPR training. This describes one local effort to improve resuscitation science education and training for medical students. Further work will need to be done to evaluate the impact of UME resuscitation curricula on medical student career choice and resuscitation outcomes.

## Introduction

Cardiac arrest is a significant health risk with approximately 350,000 people suffering a sudden cardiac arrest (SCA) per year in the United States (US) [1]. Survival from cardiac arrest has had minimal improvement over the last 30 years with overall survival rates ranging from 6.7% to 8.4% with 2015 estimates showing continued improvement equaling 10.6% [1-2]. This low survival rate has continued despite nationwide CPR teaching initiatives, dispatcher-assisted CPR programs, and increased focus on community involvement and outreach [3-5]. Japan has seen great improvement in survival and has demonstrated that instruction does help [4].

Due to the high morbidity and mortality of cardiac arrest, CPR training has been included in emergency care training for medical students throughout the US. In some areas, this has transitioned to medical students functioning as instructors and providing community CPR training [6-10]. As medical student interest in resuscitation increases, as well as the need for sufficient knowledge to train others in CPR, a structured program that is integrated into the undergraduate medical education (UME) curriculum is needed. Currently, the infrastructure to train these students is underdeveloped. Most experiences designed for medical students are brief training programs, which may not foster long-term community engagement and continued training [7, 9-10]. Though significant data exists on improving CPR performance metrics through training and debriefing [11-14], there is a paucity of knowledge on best practices and curricula for developing sustainable long-term resuscitation education programs. 

In this study, we describe a longitudinal undergraduate medical education CPR elective curriculum that was designed to promote interest and continued training in resuscitation science for medical students. Through this curriculum, medical students gain a four-year experience, including didactics on the fundamentals of resuscitation science, simulation training focused on resuscitation skills, and involvement in community CPR instruction. 

## Materials and methods

Setting and population

This is an observational program evaluation of an undergraduate medical education (UME) elective curriculum developed at the University of Arizona College of Medicine. The curriculum is a four-year longitudinal CPR elective separated into two segments: 1) preclinical (first and second-year medical school) and 2) clinical (third and fourth-year medical school). 

Problem identification and needs assessment 

As noted above, the survival from cardiac arrest is poor and is improved through the provision of bystander CPR. In the early 1990s, CPR instruction was primarily taught through basic life support classes with the endpoint being certification. In 1995, medical students at our institution identified the clear need to improve bystander CPR rates in the community. To improve rates of provision, medical students decided to increase CPR instruction through hospital-based CPR classes which were supported by the institution. Medical students received instructor certification and then taught classes in American Heart Association Basic Life Support. In 2005, the medical students wanted to increase community CPR training through chest compression-only CPR training. These students recognized their limited knowledge of resuscitation and looked for faculty support for education and training. At the institution, a significant number of faculty were focused on research and education in cardiac arrest, specifically bystander provision of CPR. The faculty had the expertise to instruct the students, support them with resources to assist in community training, and provide support for the integration of the learning objectives into the undergraduate medical education curriculum. 

To equip these learners to address this public health problem, we developed a longitudinal elective curriculum directed at resuscitation science to improve their fund of knowledge. This elective was designed to satisfy the needs of both the learner (knowledge in resuscitation) and the community environment (community CPR training). Since cardiac arrest research and education is a focus area of the institution, this is supported by local stakeholders with resources for the curriculum. 

Goals and objectives

The three primary goals of the curriculum for medical student learners were to acquire knowledge concerning resuscitation, develop skills to train others in the community, and gain experience in applying their knowledge to improve community CPR performance. The specific objectives which were defined for the longitudinal curriculum [15] include the following:

▫ To describe the importance of CPR training to individuals and the community as a whole. 

▫ To demonstrate the skills and commitment to teaching CPR through the provision of training courses in the community.

▫ To prepare the students to have an awareness of the needs of special populations regarding CPR training, including those with learning disabilities, the elderly and other high-risk groups, and children.

▫ To describe common obstacles, including the lack of access to classes, fear of infectious disease, fear of incompetence, and fear of litigation, in order to encourage more people to take CPR training.

▫ To describe the importance of key frontiers of research currently underway in cardiac arrest and CPR.

The measurable outcomes of the curriculum are:

▫ Medical students will gain knowledge in resuscitation through didactics and journal clubs (cognitive objective). A post-test and evaluation for each of the didactics and journal club sessions have been created to test the students' knowledge in resuscitation and get feedback on the sessions.

▫ Medical students will be able to demonstrate the skills needed to teach CPR to community members as directly observed during CPR classes. Students are evaluated by other students and must demonstrate proficiency in teaching the classes (cognitive objective).

▫ Medical students will be prepared in resuscitation skills through a critical appraisal at the end of the curriculum (psychomotor objective). The students have to demonstrate proficient skills in the placement of intravenous catheters, intraosseous needles, pericardiocentesis, needle chest decompression, and chest tube placement, as well as basic and advanced airway management, including bag valve mask, supraglottic airway placement, endotracheal intubation, and administration of Advanced Cardiac Life Support (ACLS) medications. The overall curriculum impact will be assessed through the number of CPR training sessions conducted, as well as the number of individuals in the community trained. 

Educational curriculum 

**Figure 1:**
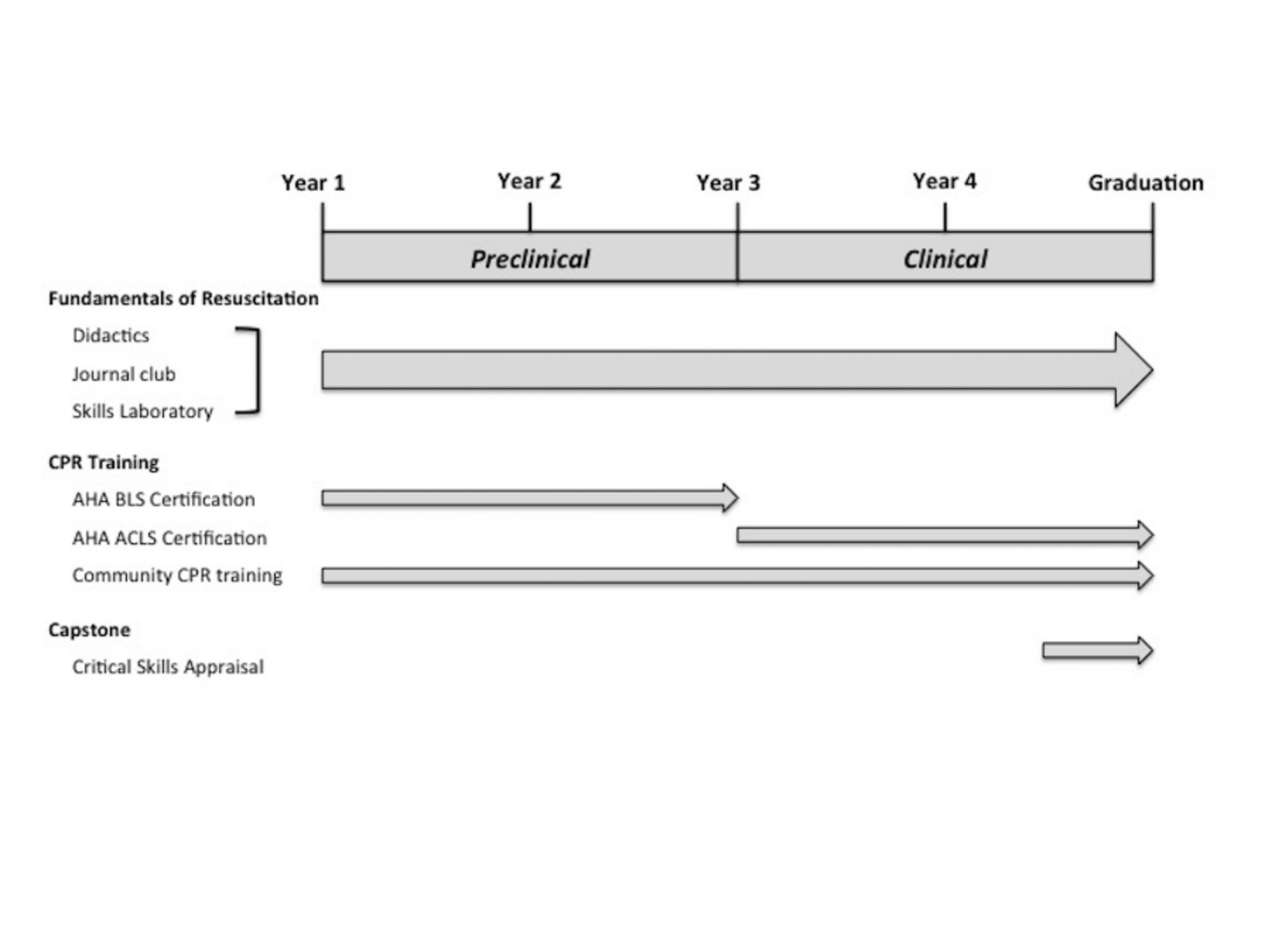
Cardiopulmonary Resuscitation (CPR) Elective Longitudinal Curriculum ACLS: Advanced Cardiac Life Support; AHA: American Heart Association; BLS: Basic Life Support

The four-year longitudinal CPR elective is divided into two segments: 1) preclinical (first and second-year medical school) [15] and 2) clinical (third and fourth-year medical school) (Figure 1) [16]. During this time period, students are provided with a structured curriculum on the fundamentals of resuscitation science, experiences in teaching CPR in the community, and a capstone critical appraisal. 

Medical student education

The fundamentals of resuscitation science training are taught through a combination of didactic lectures, journal clubs, and skills laboratory experiences occurring monthly. Bimonthly one-hour didactic sessions focus on adult and pediatric resuscitation topics, such as guideline updates, use of hypothermia, use of extracorporeal membrane oxygenation (ECMO), transfusion protocols, and others. Biannual journal club experiences are directed at the critical review of resuscitation literature, including topics such as the efficacy of epinephrine [16], chest compression-only cardiopulmonary resuscitation (CPR) and survival [3], continuous or interrupted chest compressions during CPR [17], choice of basic airway support or endotracheal intubation versus placement of a supraglottic device [18], and others. Finally, resuscitation knowledge acquisition is supplemented by bimonthly hands-on skill laboratory experiences. Medical students are instructed in emergent resuscitation procedures, including peripheral intravenous access, intraosseous access, central line placement, pericardiocentesis, chest tube placement, and airway management using direct or video laryngoscopy and supraglottic device placement.

Community education

Community CPR training is another key longitudinal aspect of the curriculum. In the preclinical segment, medical students obtain the American Heart Association (AHA) Basic Life Support (BLS) certification and are mentored in teaching community CPR by senior members of the program. Students are required to each teach 15 hours of CPR to the community. In the clinical segment, medical students obtain their ACLS certification and increase their community CPR training (20 hours). These training sessions are approximately two to three hours in length and occur throughout the community, including schools, sporting events, workout facilities, fairs, religious institutions, and retirement homes.

Finally, the critical appraisal of skills and knowledge are conducted in the simulation laboratory as a summary evaluation. This evaluation is currently conducted by two raters, the director of the CPR elective as well as an emergency medicine attending with simulation fellowship training. Students are graded on their ability to run a mock mega code. In this exercise, medical students are challenged to manage the resuscitation of a cardiac arrest patient. This is currently assessed based on student’s proficiency in CPR quality and ability to coordinate resuscitation efforts through the ACLS algorithms with a team of rescuers. An action checklist has been developed from an AHA Guideline Megacode Testing Checklist [19]. Since this is the group that writes the standards for ACLS care, their checklist is the standard of care as well. Feedback obtained regarding this elective includes seven questions: 

1) What did you find most useful during this resuscitation elective?

2) What was most challenging about this elective?

3) What improvements could be made to this elective?

4) Regarding the didactic portion of this elective, what feedback do you have?

5) Regarding the journal clubs you attended, how effective did you find these to be for your learning of resuscitation science? 

6) Regarding the hands-on skills training and simulation lab time, what feedback do you have?

7) Please rate your comfort level with teaching CPR to community members after completion of this elective.

These were all measured using a Likert scale with 1 representing highly uncomfortable, 3 for neutral, and 5 for highly comfortable. 

There is also a leadership skills section added to the checklist that focuses on leadership in the code setting (Table 1). 

**Table 1:** Checklist for Student Code Evaluation Adapted from the American Heart Association Megacode Testing Checklist [19] BVM: bag valve mask; CPR: cardiopulmonary resuscitation; IO: intraosseous; IV: intravenous; J: joules; VF: ventricular fibrillation; VT: ventricular tachycardia

ACTION	PASS	NEEDS REMEDIATION
Assess for patient responsiveness (< 10 sec)		
Call for help - activate code blue		
Check for a femoral or carotid pulse and correctly identify that the patient does not have a pulse (< 10 sec)		
Start adequate chest compressions		
Appropriate airway management with BVM		
Place defibrillator pads on the patient, turn Lifepack on		
Establish IV access or IO if IV unsuccessful		
Give 1 mg epinephrine 1:10,000 IV/IO		
Perform pulse and rhythm check after 2 minutes of CPR		
Identify rhythm; if VT or VF, clear, charge, and defibrillate at 200 J		
Immediately resume CPR		
LEADERSHIP SKILLS		
Ensures high-quality CPR at all times		
Assigns team member roles		
Ensures that team members perform well		

Medical students who demonstrated completion of the above work associated with the longitudinal CPR elective are then awarded a College of Medicine elective credit which satisfies two weeks of their fourth year of medical school. 

CPR community training

Medical students taught either of two classes: the American Heart Association (AHA) Basic Life Support course or a bystander chest compression only (CCO) CPR course. Training for AHA CPR was done utilizing the standardized programs from the AHA. Participants did receive AHA certification if they attended the AHA-approved course. Concerning CCO CPR, there are no specific formalized training courses which have been developed. Therefore, to assure quality teaching, students developed a standardized CCO CPR training program which led to a PowerPoint® (Microsoft® Corp., Redmond, WA) lecture for the training events. The developed educational content was evaluated and approved by the Sarver Heart Center Cardiopulmonary Resuscitation Research Group. 

The CPR training was held at venues throughout Arizona. Training was commonly conducted in schools (primary or secondary schools), sporting events, and local community events. 

Implementation

The program was launched at the medical school after approval of the curriculum by the Institutions Curriculum Committee of the University of Arizona. The program was accepted as the first longitudinal curriculum for the medical school. Program implementation was facilitated through assistance from the Sarver Heart Center and the Department of Emergency Medicine. The faculty mentor of the program was from the Department of Emergency Medicine. Faculty from the Division of Cardiology at The Sarver Heart Center assisted in didactics and skills training. Facility use, including the skills lab space, was supported from the College of Medicine. Funding for CPR manikins and equipment for community training was provided by the Sarver Heart Center and the College of Medicine. Finally, administrative support of a curriculum director was provided by the College of Medicine, as well as internet and web development support. 

Program evaluation

Medical students access all learning objectives and materials through an online website where student progress was tracked through the four years of the curriculum (2010 to the present) [15]. The online database system tabulated attendance at didactics, journal clubs, and skills labs, as well as the CPR classes taught in the community. Didactic lectures were also available through the online systems to allow asynchronous education. Grading was through the performance of the required aspects of the curriculum. 

Cognitive outcome measures of the learners were assessed using the online database system, as well as directed student observations by faculty. The psychomotor outcome measure was assessed by faculty at the monthly simulation events which were tracked in the online system. This included demonstrating proficiency in placing peripheral IV’s, intraosseous needle placement, central line placement, needle decompression of the chest, and chest tube placement, as well as pericardiocentesis. These skills were also included in the final summative evaluation of the resuscitation skills appraisal.

Program performance was assessed from data concerning medical student enrollment in the program, CPR training events conducted, venues of events, and the number of individuals trained. 

## Results

Program involvement

The number of medical student enrollments in the elective from 2010 - 2015 is noted in Table 2. During this time period, the total number of students enrolled in the CPR longitudinal elective was 186 students with approximately 37 students per year. Considering the total number of enrolled medical students (n = 2,216), 8.4% of these students participated in the CPR elective during this period. 

**Table 2:** Enrollment in the Cardiopulmonary Resuscitation (CPR) Elective from 2010 to 2015 Data are presented as total enrolled students in the two phases of the program (preclinical and clinical).

Year	Total Students Enrolled in Elective Per Year	Enrolled Medical Student Class (All 4 Years)	Percent of Total Student Population in CPR Elective
2010 - 2011	40	440	9.1%
2011 - 2012	35	440	8.0%
2012 - 2013	43	450	9.6%
2013 - 2014	39	446	8.7%
2014 - 2015	29	440	6.6%
Total	186	2,216	8.4%

Community outreach

A key aspect of the CPR elective is community outreach. Table 3 notes the number of individuals trained through the CPR elective from 2010 to 2015. The total number of individuals trained over this period was 22,694 with 491 total training events held. The average number of individuals trained per event was approximately 45. 

**Table 3:** Number of Trainings Held and People Trained in Bystander CPR by Medical Students in the Longitudinal Elective from 2010-2015 CPR: cardiopulmonary resuscitation

Academic Year	CPR Trainings Taught	People Trained in Bystander CPR	People Trained Per Program
2010 - 2011	92	3,652	40
2011 - 2012	123	7,424	60
2012 - 2013	106	4,227	40
2013 – 2014	95	4,068	43
2014 - 2015	75	3,323	44
Total	491	22,694	46

Medical students taught either of two classes: the American Heart Association Basic Life Support course or a bystander chest compression only (CCO) CPR course. Training for AHA CPR was done utilizing the standardized programs from the AHA. Concerning CCO CPR, there are no specific formalized training courses that have been developed. Therefore, to assure quality teaching, students developed a standardized CCO CPR training program, which led to a Powerpoint lecture for training events. The developed educational content was evaluated and approved by the Sarver Heart Center Cardiopulmonary Resuscitation Research Group. 

The scheduling and coordinating of the training program was managed by the medical student interest group called Resuscitation Education and CPR Training (REACT). This is the group from which the longitudinal elective was originally developed. To manage a large amount of planning and coordination, the REACT group structure includes formalized leadership to respond to these needs, including a president, vice-president, secretary, treasurer, community outreach coordinator, research program coordinator, media relations, AHA CPR representative, and a university outreach coordinator.

The CPR training courses were held at venues throughout Arizona. The training courses were commonly conducted in schools (primary or secondary schools), sporting events, and local community events. As an example of the diverse training classes held by the medical students, a complete list of the training events held in 2013 is noted in Table 4 ranging from elderly retirement communities [20] to recreational vehicle (RV) parks and festivals. More recently, larger community events have involved the use of ultra-brief videos for exposure of large populations to CPR training (Abstract 495: Goldberg L, Panchal B, Sanders AB, Panchal AR: Evaluation of bystander perceptions of CPR following a statewide chest-compression-only CPR initiative. Presented at the Society of Academic Emergency Medicine Ann. Mtg., May 14-17, 2014 Dallas, TX) [6, 21-23].

**Table 4:** Cardiopulmonary Resuscitation (CPR) Training Session Venues List of venues where CPR training sessions were conducted, type of event, and the number of individuals exposed to CPR training or were directly trained at the events. *: exposed to CPR training through ultra-brief video. 4-H: Head, Heart, Hands, and Health; JOLT: Journey: Opportunities for Leaders of Tomorrow; RV: recreational vehicle

Venue	Type of Event	Number Trained
University of Arizona Basketball	Sporting Event	14,000*
Tucson Festival of Books	Community Event	1,000*
Gootter Grand Slam	Cardiac Arrest Charity Event	650*
University High School Boost Programs	School Education Program	310
Desert Sky Middle School	School Education Program	300
University of Arizona Graduate Assistant Training	School Education Program	250
Cochise College	College Education Program	200
Gootter Gala	Cardiac Arrest Charity Event	200
Career Fest at Cholla High School	School Education Program	175
4-H JOLT Camp	Summer Camp Program	150
Voyager RV Resort	Community Event	138
Rancho Vistoso (Mackstaller)	Community Event	132
Children’s Museum Tucson	Community Event	115
Beyond Tucson	Community Event	103
Amphi High School Teachers	School Education Program	80
Apollo Middle School	School Education Program	75
Cyclovia	Community Event	75
St. Thomas Marian Club	Community Event	50
Splendido Health and Wellness	Community Event	50
Vail Academy and High School	School Education Program	50

Research

Research production was originally included as a curriculum requirement for the CPR elective. However, as the interest group and longitudinal curriculum developed, students became more interested in questions surrounding the provision of CPR. Through the CPR elective, the medical students learned the science behind resuscitation and then became interested in methods to improve CPR education. With the assistance of their elective faculty mentors, medical students began the process of a generation of research questions and developed plans to test these concepts. 

The students have been involved in the planning, development, and coordination of multiple research studies. The students have been pivotal in the development of projects leading to several abstracts (Abstract: Thiede R: Impact of finding a chest compression only ultra-brief video at a mass gathering sport event on lay bystander CPR performance. Society of Academic Emergency Medicine, San Diego, CA, May 12-15, 2015; Poster 260: Beskind DL, Thiede R, Hoyer R, et al.: Impact of viewing an ultra-brief chest compression only (CCO) video or teaching a CCO-CPR class on lay bystander CPR performance in a high school. AHA Resuscitation Science Symp., Chicago, Nov. 16, 2014, http://aha.scientificposters.com/epsAbstractAHA.cfm?id=1; Abstract 495: Goldberg L, Panchal B, Sanders AB, Panchal AR: Evaluation of bystander perceptions of CPR following a statewide chest-compression-only CPR initiative. Presented at the Society of Academic Emergency Medicine Ann. Mtg., Dallas, TX, May 14-17, 2014) and peer-reviewed publications [6, 21-23].

## Discussion

This curriculum development describes one local effort to improve resuscitation science training for young physicians through a longitudinal CPR elective curriculum. Medical students enrolled in the longitudinal CPR elective participated in a comprehensive curriculum on resuscitation science linking medical knowledge to a practical literature review and skills enhancement (Figure 1). Over the program evaluation period, the elective enrolled 186 medical students, accounting for approximately 8% of the total medical student population at the institution. Further, the enrolled medical students were able to train over 22,000 community members in CPR during this period (Table 2). Thus, the CPR elective was able to improve the resuscitation knowledge of a large number of young physicians while serving the community through CPR education at the same time. Most surprisingly, as these medical students became more interested in resuscitation science, many became involved in research studies to address questions concerning resuscitation education. 

This curriculum is one example of a program designed to encourage medical students to teach CPR [7-9, 24-26]. Commonly, medical students teach CPR in the community and, in return, gain training in resuscitation science as noted in improved knowledge and practical skills [7, 10, 26]. However, their experience with resuscitation science was limited to these training experiences and often is not part of a developed UME curriculum. Other institutions have integrated teaching CPR as part of their problem-based learning curriculum or in the emergency medicine coursework [8, 10]. These experiences focused on teaching basic CPR skills without a structured resuscitation science curriculum. This longitudinal CPR elective differs from those in that the structured UME curriculum is designed to develop resuscitation science knowledge and skills and enhances the didactic experiences through community CPR training. 

This is the first study to demonstrate the implementation of a robust UME curriculum in resuscitation science with sustained medical student enrollment, along with consistent community CPR training. This longitudinal curriculum may provide a framework for other institutions to develop resuscitation electives to promote interest and involvement in resuscitation in young physicians. Furthermore, the program also provides medical students with experiences that may not be common, given in the standard UME curriculums (e.g., journal clubs and skills laboratory experiences), and facilitates important patient-centered communication experiences necessary for physician development [7].

Limitations

This program was developed at one institution, which limits its implementation. Development of similar programs in other environments may not have the same outcomes as noted in this evaluation without a strong environment of medical student interest groups. In addition, other programs may not have simulation center availability, faculty oversight, or funding, which may limit the generalizability. However, significant data does exist which indicates that medical students are readily able to teach CPR and benefit educationally from these teaching experiences [7-10, 24-26]. Furthermore, other programs that have integrated CPR training into their curriculum support the feasibility of this curriculum in other settings [8-10].

There is a possibility of inaccuracy in the tabulated data since the training numbers were self-reported by the students. However, the students were unaware of this curriculum evaluation, and there was no minimum requirement for numbers of individuals trained by students enrolled in the curriculum. Students were only required to account for their hours spent training the community. 

Further, although medical students were able to satisfy curriculum requirements and all passed the summative appraisal, data was not collected concerning student impressions concerning the achievement of curricular objectives. Future evaluations will focus on student feedback on the learning sessions, objective achievement in terms of their actions during a mock code, and assessment of their leadership skills, as well as their comfort with community training. Based on this, we have implemented evaluation forms for all of the didactic and skills sessions moving forward to be able to critically appraise the sessions to allow for continued curriculum improvements.

This evaluation was also not able to assess the impact of the longitudinal curriculum on the overall community and hospital CPR performance. No hospital data has been gathered concerning the impact of students completing a longitudinal CPR elective. However, previous studies conducted by students in the curriculum have demonstrated the positive impact of ultra-brief video community CPR instruction in a shopping mall [21], high school [6], and mass gathering events [22-23]. This suggests that community CPR training classes by medical students is beneficial and may impact mortality from out-of-hospital cardiac arrest (OHCA). Of note, training sessions conducted through these medical students have yielded bystander performance of CPR, along with survival from OHCA [25].

There was a minimal assessment of knowledge and skills retention of the community members taught CPR by the students. Therefore, it is difficult to assess the impact. However, we do know of two people in the community who were taught CCO-CPR by the students that successfully resuscitated two people who experienced OHCA and both survived, neurologically intact. 

## Conclusions

Implementation of a longitudinal CPR elective exposed medical students to resuscitation science education and fostered community CPR training. This evaluation, which met IRB exemption status, describes the development of a longitudinal CPR elective curriculum for undergraduate medical education. Further work will need to be done to evaluate the impact of UME resuscitation curricula on student perceptions and community CPR performance and outcomes.
